# Study on the functional role of immunoglobulin E as surrogate marker for HIV/AIDS infection

**DOI:** 10.1186/1742-4690-9-S2-P96

**Published:** 2012-09-13

**Authors:** B Sonaimuthu, V Baghyanathan

**Affiliations:** 1 King Institute of Preventive Medicine & Research, Chennai, India

## Background

IgE class of antibodies has been found in mammals and plays an important role in allergic and hypersensitivity reactions. Certain viral infections are known to produce specific IgE antibodies, to the extent that significant changes in the level of total serum IgE may occur. Study attempts to associate the Level of Ig E in HIV progression.

## Methods

The study involves fifty HIV seropositive patients attending Anti-Retroviral Therapy Centre, Department of Sexually Transmitted Disease, Rajaji Government Hospital, and Madurai,India subjected for the present study. The individual involves 27 HIV/ AIDS Male patients, 23 HIV/ AIDS Female patients. The control sample comprises 15 HIV sero negatives. The samples were collected at the informed consent of the patients. Serum sample were collected and IgE was quantified using MAGIWELL IgE quantitative solid phase Enzyme- linked Immunosorbent assay (ELISA).

## Results

The study documents highest percentage of deviation from the control observed in Male HIV seropositives (43.7%) and age-wise influence documents highest percentage of deviation in the age group 15- 29 years (56%).

## Conclusion

Serum IgE level in the present study found to be elevated from the normal range documents the existence of imbalance between Th 1 and Th 2 and associated with T-cell dysfunction and a hypergammaglobulinemia. The present results suggest that elevation of circulating IgE levels may be due, at least in part, to specific IgE directed to the HIV virus rather than as a result of a nonspecific phenomenon. HIV infected adults indicate that total IgE is also increased during the early stages of disease, and this elevation appears to be independent of CD4 counts and is not correlated with the levels of other immunoglobulins, suggesting an important role for IgE as a surrogate marker of disease progression Further research need to be exploited to bring out the exact role of IgE in HIV pathogenesis.

